# Cardiac Cell Exposure to Electromagnetic Fields: Focus on Oxdative Stress and Apoptosis

**DOI:** 10.3390/biomedicines10050929

**Published:** 2022-04-19

**Authors:** Ilenia Martinelli, Mathieu Cinato, Sokhna Keita, Dimitri Marsal, Valentin Antoszewski, Junwu Tao, Oksana Kunduzova

**Affiliations:** 1 National Institute of Health and Medical Research (INSERM) U1297, CEDEX 4, 31432 Toulouse, France; ilenia.martinelli@unicam.it (I.M.); cinato.mathieu@aol.fr (M.C.); sokhna.keita@inserm.fr (S.K.); dimitri.marsal@inserm.fr (D.M.); 2 Unité Mixte de Recherche (UMR) 1297, Université Toulouse III Paul-Sabatier, 31062 Toulouse, France; valentin.antoszewski@univ-tlse3.fr; 3 Laboratoire Plasma et Conversion d’Energie (LAPLACE), National Polytechnic Institute of Toulouse, The Ecole Nationale Supérieure d’Electrotechnique, d’Electronique, d’Informatique, d’Hydraulique et des Télécommunications (ENSEEIHT), Toulouse University III, 31071 Toulouse, France; tao@laplace.univ-tlse.fr

**Keywords:** electromagnetic fields, cardiomyoblasts, oxidative stress, apoptosis

## Abstract

Exposure to electromagnetic fields (EMFs) is a sensitive research topic. Despite extensive research, to date there is no evidence to conclude that exposure to EMFs influences the cardiovascular system. In the present study, we examined whether 915 MHz EMF exposure affects myocardial antioxidative and apoptotic status in vitro and in vivo. No statistically significant difference in the apoptotic cell profile and antioxidant capacity was observed between controls and short-term EMF-exposed mouse cardiomyocytes and H9C2 cardiomyoblasts. Compared with sham-exposed controls, mice subjected to a 915 MHz EMF for 48 h and 72 h had no significant effect on structural tissue integrity and myocardial expression of apoptosis and antioxidant genes. Therefore, these results indicate that short-term exposure to EMF in cardiac cells and tissues did not translate into a significant effect on the myocardial antioxidant defense system and apoptotic cell death.

## 1. Introduction

Human exposure to electric and magnetic fields (EMFs) is an integral part of modern societies. Sources of exposure to EMFs are largely varied and increasingly prevalent. The EMFs generate the electromagnetic waves of different frequencies from natural environments, such as the solar energy and geomagnetic field, or from man-made sources, including transmission towers, telecommunications, home appliances, mobile phones, Wi-Fi, and base stations [1,2]. A number of studies have reported that EMF exposure of living systems can affect vital cellular processes [3,4,5]. However, the massive findings originated from in vitro and in vivo studies remain controversial. The lack of sufficient scientific data on EMF-exposed animal models has led to diverse apprehensions [6,7,8,9].

EMFs coupled with biological systems depend on the frequency ranges of the employed signals, which are classified as extremely low frequency field(s) (ELF, between 1  and 100 kHz) and high frequency (HF) fields, in the band of the radio frequency fields (RF, 100 kHz–3 GHz), and of the microwaves (MW, above 3 GHz) [10,11]. Numerous in vitro and in vivo studies have been performed in order to dissect the impact of EMFs generated by mobile phones and their base station in the range of 890–960 MHz. Several reports suggest that exposure to 900 MHz EMFs emitted by a mobile phone may cause cell apoptosis and oxidative stress in kidney, endometrium, and brain tissue [12,13,14,15,16]. Multiple molecular mechanisms have been proposed to explain the observed cellular effects of EMFs in the biological system. It has been claimed that possible effects could be triggered by generation of reactive oxygen species (ROS), disturbance of DNA-repair processes, and ROS-associated signaling pathways. In this scenario, the balance between pro- and antioxidant enzymes can be upset by an increase in ROS or by a decrease in antioxidant enzymes, including superoxide dismutase (SOD), catalase, and glutathione peroxidase (GSH-Px) [1,17].

Cardiac cells are particularly sensitive to changes in antioxidant capacity and ROS status due to the high energy metabolism in mitochondria [18]. Mitochondria, the main ROS generators, are abundant in heart tissue, constituting 40% of the cell volume of adult cardiac cells [19]. ROS are generated at an accelerated rate in the failing heart, suggesting that oxidative stress is common in cardiac cell dysfunction. Excessive ROS formation in cells has been shown to trigger the intrinsic apoptotic pathways via multiple mechanisms, including Bcl-2-associated X protein (Bax) translocation to the mitochondria, release of cytochrome c, and caspase activation [18]. Due to the impairment of transcriptional responses to oxidative stress and the decreased expression of antioxidant defense enzymes, oxidative stress was demonstrated to increase in damaged heart tissue [18]. While excessive accumulation of ROS-mediated damage is widely accepted as one of the primary causes of cardiac cell dysfunction, ROS also act in signaling pathways [19].

Given the ubiquitous nature of EMFs and their widespread applications, conclusive investigations into the potential effects of EMFs on cardiac cells are critical. However, there are many unknowns related to the influence of EMFs on antioxidant potential and cell death status in the heart. In this study, we examined the short-term exposure to 915 MHz EMFs on myocardial antioxidant capacity and apoptotic cell death in vitro and in vivo. Since morphological and biochemical analyses are the most applied methods to detect apoptotic cell death after EMF exposure [20], here, we examined the messenger ribonucleic acid (mRNA) levels of key apoptosis-related genes *Bcl2*, *Bax*, *caspase-3*, and *caspase-8* [21]. Among endogenous defense mechanisms responsible for the EMF-induced effects, catalase and SOD are crucial antioxidative enzymes that have been implicated in human diseases [22]. We also determined the expression levels of antioxidant defenses, including *catalase* and mitochondrial *SOD* in cardiac cells and tissues exposed to EMFs.

## 2. Materials and Methods

### 2.1. In Vitro Studies

Rat cardiomyoblasts (H9C2 cell line) were maintained in a growth medium with Dulbecco’s Modified Eagle Medium (DMEM) Glutamax supplemented with 10% fetal bovine serum and 1% penicillin–streptomycin at 37 °C in 5% CO_2_. The cultured H9C2 cells were grown to 80–90% in a Petri dish and were exposed to EMFs for 24 h, 48 h, and 72 h. Cardiac myocytes were isolated from male 10-week-old C57BL6 mice using the method described by Xu et al. [23]. Briefly, hearts were perfused by the Langendorff method with HEPES-buffered Earle’s balanced salt solution (GIBCO-BRL) supplemented with 6 mM glucose, amino acids and vitamins (buffer A), and then with buffer A containing 0.8 mg/mL collagenase B (Boehringer-Mannheim, Mannheim, Germany) and 10 μM CaCl_2_. The enzyme solutions were filtered (2-μm pores) and recirculated through the heart until the flow rate doubled (12–20 min); the left ventricle was then removed and minced in collagenase containing buffer A. Thereafter, the tissue pieces were transferred to fresh (enzyme free) buffer A supplemented with 1.25 mg/mL taurine, 5 mg/mL BSA (Sigma-Aldrich, St. Louis, MO, USA), and 150 μM CaCl_2_ and mechanically dissociated by gentle trituration. The resulting suspension was filtered and isolated cells were obtained by sedimentation. The cultured primary cardiomyocytes were exposed to 915 MHz EMFs for 1 h, 3 h, and 24 h.

#### 2.1.1. Radiofrequency Equipment to In Vitro Studies

To evaluate the biological effects of radiofrequency EMF on living cardiac cells, we used a transverse electromagnetic (TEM) cell (FCC-TEM-JM1 from Fischer Custom Communications, Torrance, CA, USA). A solid state radiofrequency generator (2022D from Marconi Instruments Limited, Saint Albans, UK) was used to power the TEM cell. In order to define dielectric property of the biological medium, the technique of measuring samples reported on a microstrip transmission line was used [24]. The TEM cell used in an in vitro experiment was modeled on HFSS, as described previously [24].

#### 2.1.2. In Vitro EMF Exposition Parameters

In vitro cell culture experiments were conducted in a standard cell culture incubator at 37 °C, 5% CO_2_, and at 21% O_2_ levels. Control cells were treated in the same manner as the exposed ones but were not subjected to EMFs at any point. Using Marconi 2022D (Marconi Instruments Limited, Saint Albans, UK) tune without modulation (for the continuous wave mode), the 20 mW power level led to a maximum local specific absorption rate (SAR) of ~0.08 W/kg in cultured cells. The simulation was performed using high frequency structure simulator (HFSS) software version 15. The 20 mW corresponded to the highest output power of the generator. The TEM cell was placed inside a cell culture incubator. The power supply of the TEM cell was carried out by a coaxial cable through an orifice for the passage of pipes.

### 2.2. In Vivo Studies

#### 2.2.1. Animals

The investigation conforms to the Guide for the Care and Use of Laboratory Animals published by the US National Institutes of Health (NIH Publication no. 85-23, revised 1985) and was performed in accordance with the recommendations of the French Accreditation of the Laboratory Animal Care (approved by the local Centre National de la Recherche Scientifique ethics committee). The RjOrl:Swiss female mice at 6 months of age were used for these investigations (Envigo RMS, Gannat, France). After arrival, animals were randomized and housed in groups of four in polycarbonate cages, enriched with paper. Mice were allowed free access to standard food pellets and tap water. Temperature was controlled at 21 ± 2 °C. Two days prior to EMF exposure, mice were adapted to new environmental conditions in a room in which, subsequently, the whole experiment was performed. The light was on a 12 h light–12 h dark cycle, with light on at 8 am. Mice were then randomly segregated into two groups (control, *n* = 6 and EMFs, *n* = 10) and exposed to a 915 MHz EMF for 48 h and 72 h. Control animals were treated in the same manner but were not subjected to EMFs at any point.

#### 2.2.2. Radiofrequency Equipment to In Vivo Studies

**Figure 1:**
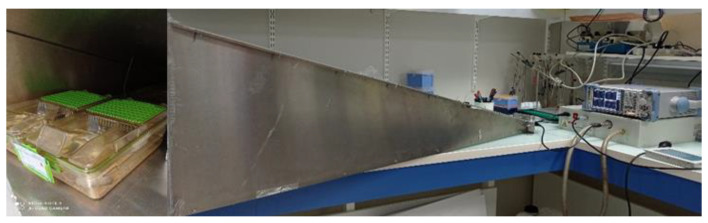
GTEM cell to explore in vivo studies.

In vivo experiments were performed in a Giga-TEM (GTEM) cell to accommodate cages (up to 4) in which the animals exposed to EMFs. The model of the GTEM cell is shown in Figure 1. Twenty-four hours before the experiment, the mice were placed in the GTEM to adaptation.

We used a solid-state radiofrequency generator of a fixed 915 MHz frequency (WSPS-915–1000) (Chengdu Wattsine Electronics Technology, Chengdu, Sichuan, China). A full-wave electromagnetic simulation was taken to verify if matched load conditions with one or several cages could be obtained by center conductor tuning [25]. In these simulations, the webster was modeled by the sphere of equivalent volume. Three spheres were placed along the wave propagation axis at the end of the GTEM cell. By using a relative permittivity of 55 and equivalent conductivity of 3 S/m, the estimated SAR values reached ~40 W/kg. The calculation of whole body SAR was taken by volume integration of absorption power using MATLAB codes. The field data were taken from HFSS simulation exportation. The input RF power was 4 W in the HFSS simulation and in the experimentation.

### 2.3. Quantitative RT–PCR Analysis

The expression of genes was assessed using quantitative polymerase chain reaction (qRT-PCR). Total RNAs were isolated from the rat cardiomyoblast cell line H9C2, primary cardiomyocytes, and cardiac tissue using the RNeasy mini kit (Qiagen, Hilden, Germany). Total RNAs (300 ng) were reverse transcribed as previously described [26]. Primer sequences were detailed in Supplementary Table S1. The expression of target mRNA was normalized to GAPDH and RPLP0 mRNA expression.

### 2.4. Morphology

Heart tissue integrity was assessed by hematoxylin and eosin (H&E) stain, according to standard methods. In both the experimental group and the control group, mice were sacrificed to collect hearts at 48 h and 72 h. Tissues were collected under a protocol approved by the Animal Care and Use Committee of INSERM. Tissues were snap frozen for further analyses. In cardiac tissue, the H&E stain was performed to evaluate morphological features, including damaged myofibers, collagen accumulation, necrotic myofibers, and myocyte size.

### 2.5. TUNEL Assay

The apoptosis level was assessed using the DeadEndFluorometric TUNEL system according to the manufacturer’s instructions (Promega, Madison, WI, USA). TUNEL staining were performed in heart cryosections to detect nuclear DNA fragmentation during apoptosis.

### 2.6. Western Blot

Proteins from cardiac tissues were extracted using the RIPA buffer and quantified using the Bio-Rad Protein Assay (Bio-Rad, Hercules, CA, USA). Proteins were resolved by SDS-PAGE and western blotting. Immunoreactive bands were detected by chemiluminescence with the Clarity Western ECL Substrate (Bio-Rad, Hercules, CA, USA) on a ChemiDoc MP Acquisition system (Bio-Rad, Hercules, CA, USA), as previously described [26]. Antibodies used in this study were SOD2 and tubulin; both were purchased from Santa Cruz Biotechnology (Santa Cruz, CA, USA). Tubulin was used as a loading control.

### 2.7. Statistical Analysis

Data are expressed as mean ± SEM. A statistical comparison between the two groups was performed by Student’s *t*-test and the ANOVA test was used to assess the statistical significance of the difference between more than two study group means. The Kruskal–Wallis test was used to assess the statistical significance of the difference of a non-parametric variable between more than the two study groups using GraphPad Prism version 5.00 (GraphPad Software, Inc., San Diego, CA, USA). *p*-values < 0.05 were considered to indicate statistically significant differences.

## 3. Results

**Figure 2:**
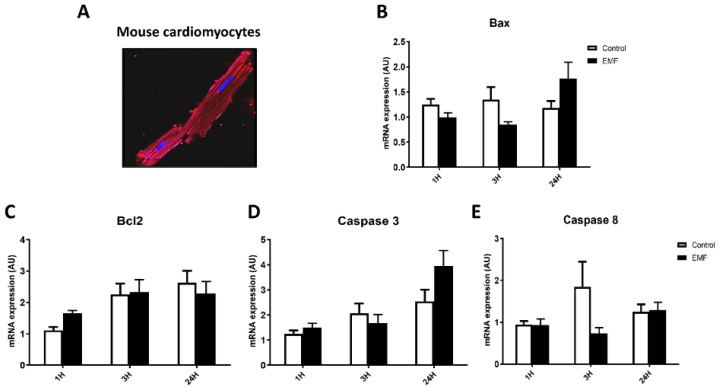
Apoptosis-related gene expression in mouse cardiomyocytes exposed to EMFs. Representative pictures of cultured mouse cardiomyocytes (**A**) and mRNA expression levels of *Bax* (**B**), *Bcl2* (**C**), *caspase 3* (**D**), and *caspase 8* (**E**) in cardiomyocytes exposed to 915 MHz EMFs for 1 h, 3 h, and 24 h. The results present the mean ± SEM of three independent experiments.

In order to determine whether radiofrequency EMFs affect the antioxidant defense system and apoptosis at the cellular level, adult mouse cardiomyocytes were exposed to 915 MHz EMF at 13 dBm. Gene expression profiles of apoptosis-related factors, including *Bax*, *Bcl2*, *caspase 3*, and *caspase 8*, were determined in cultured cardiomyocytes exposed to EMFs for 1 h, 3 h, and 24 h. As shown in Figure 2, exposure of myocytes to a 915 MHz did not significantly change the mRNA expression of *Bax*, *Bcl2*, *caspase 3*, and *caspase 8* at 1 h and 3 h as compared to control conditions. In exposed cells for 24 h, *Bax* and *caspase 3* mRNA levels showed a tendency to increase, but this did not reach statistical significance (Figure 2B,D).

**Figure 3:**
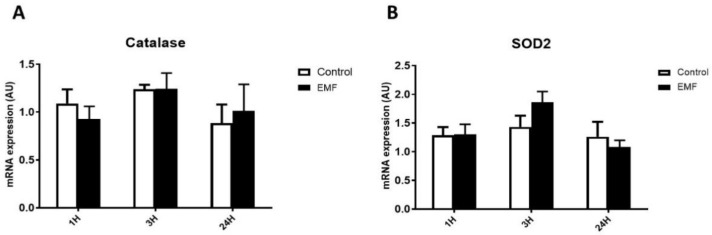
Effects of 915 MHz EMFs on antioxidant enzyme gene expression in cultured cardiomyocytes. Quantitative analysis of the mRNA expression levels of *catalase* (**A**) and *SOD2* (**B**) in cardiomyocytes exposed to EMFs for 1 h, 3 h, and 24 h. The results present the mean ± SEM of three independent experiments.

To evaluate the level of antioxidant capacity in cardiac cells in conditions of the electromagnetic field, we next examined the expression of *catalase* and *SOD2* genes in cardiomyocytes subjected to a 915 MHz frequency EMF for 1 h, 3 h, and 24 h. As shown in Figure 3, real-time qPCR analysis revealed no differences for expression levels of *catalase* and *SOD2* between control and EMF-exposed cardiomyocytes.

These results suggest an adaptive response of cells subjected to a 915 MHz continuous wave radiofrequency exposure for 1 h, 3 h, and 24 h.

**Figure 4:**
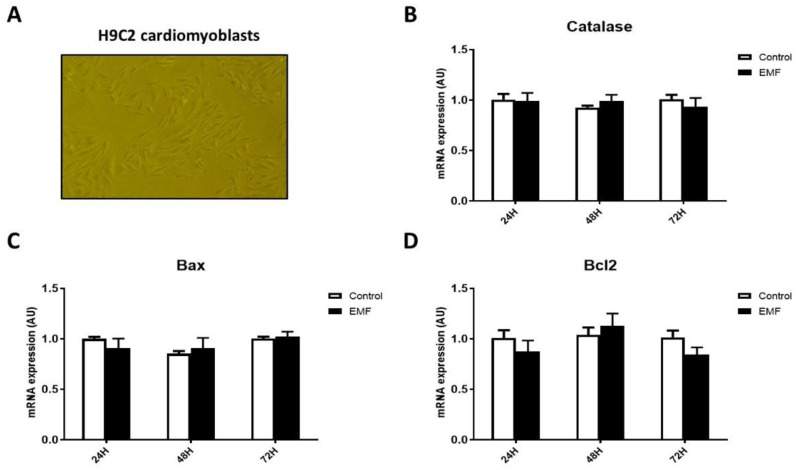
Gene expression profile of antioxidant enzyme *catalase* and pro- and anti-apoptotic factors in H9C2 cells exposed to radiofrequency EMFs at 915 MHz for 24 h, 48 h, and 72 h. Representative pictures of H9C2 cardiomyoblasts (**A**) and mRNA expression levels of *catalase* (**B**), *Bax* (**C**), *Bcl2* (**D**). The results present the mean ± SEM of three independent experiments.

Interestingly, similar results were obtained in H9C2 cardiomyoblasts. As shown in Figure 4, no significant differences were observed in the expression levels of apoptosis-related factors and antioxidant enzyme *catalase* in cells subjected to EMFs for 24 h, 48 h, and 72 h. These data suggest that short-term exposition to EMFs in cardiac cells did not translate into a significant effect on antioxidant enzymes and apoptotic cell death.

**Figure 5:**
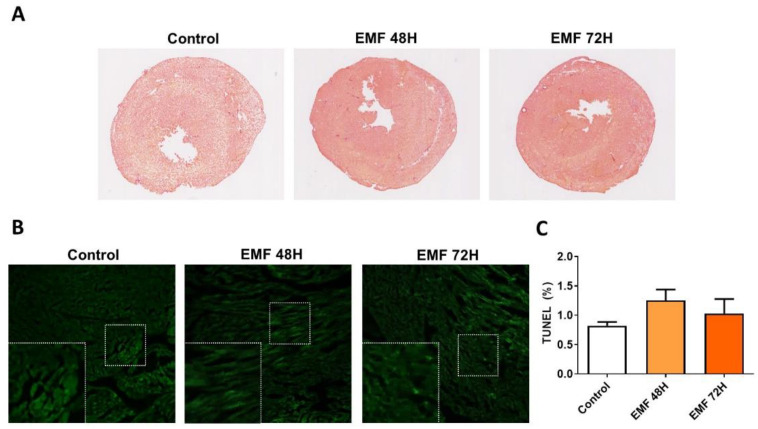
Effects of 915 MHz EMFs on cardiac structure and apoptosis in mice exposed for 48 h and 72 h. Representative images of cardiac sections stained with H&E (**A**). Scale bar: 2.5 mm. Representative images of cardiac sections stained with TUNEL (**B**) and quantification (**C**). Scale bar: 100 µm. The results present the mean ± SEM, *n* = 3.

In order to evaluate in vivo effects of EMFs in mice, we next examined whether exposition of mice to EMFs affected structural integrity, apoptosis, and antioxidant capacity in cardiac tissues. As shown in Figure 5A, no significant differences between EMF and control groups were observed at the structural level in terms of collagen accumulation, necrotic myofibers, and myocyte size. We next examined whether electromagnetic stress could cause cardiac apoptosis after mice were exposed to 915 MHz for 48 h and 72 h. As shown in Figure 5B,C, both control mice and EMF-exposed mice exhibited minimal apoptosis assessed by TUNEL staining in the left ventricles.

**Figure 6:**
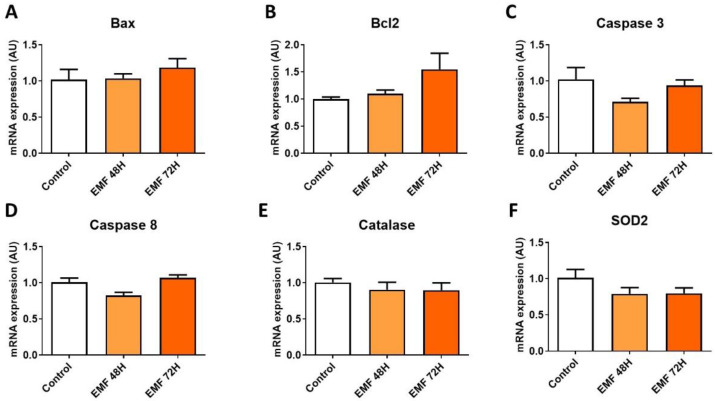
Gene expression profile of antioxidant enzymes and apoptosis-related factors in mice subjected to EMFs. Quantitative analysis of the mRNA expression levels of *Bax* (**A**), *Bcl2* (**B**), *caspase 3* (**C**), *caspase 8* (**D**), *catalase* (**E**), and *SOD2* (**F**) in mice exposed to 915 MHz EMFs for 48 h and 72 h and control animals. The results present the mean ± SEM, *n* = 6–10.

Furthermore, no significant differences were found in the expression of genes involved in the antioxidant system and apoptosis regulation in cardiac tissue from mice exposed to 915 MHz EMFs and control animals (Figure 6).

**Figure 7:**
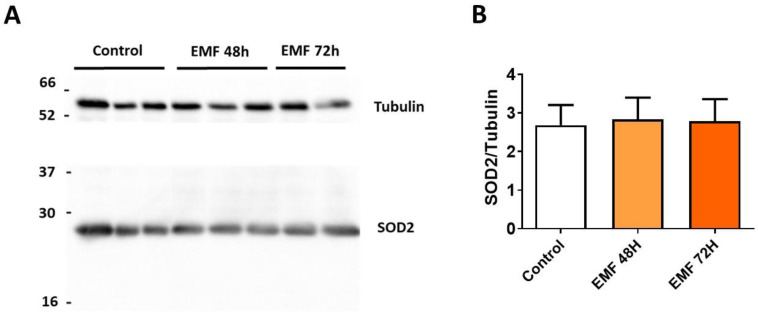
Myocardial expression of SOD2 in mice exposed to 915 MHz EMFs. Representative western blot image (**A**) and quantification (**B**) of SOD2 protein expression levels in mice exposed to 915 MHz EMFs for 48 h and 72 h and control animals The results present the mean ± SEM, *n* = 6–10.

Western blot analysis further confirmed no significant changes in the expression of antioxidant enzyme SOD2 in cardiac tissues from mice subjected to 915 MHz for 48 h and 72 h as compared to control mice (Figure 7).

## 4. Discussion

The use of mobile phones and base stations are increasing worldwide [27]; however, the biological effects of EMF exposure on cardiac cells and tissues remain unclear. Cardiomyocytes are sensitive to a wide range of internal and external stimuli linked to ROS formation [18]. Oxidative stress and cell apoptotic reprogramming are largely involved in the pathophysiology of human diseases, where both survival and cell death signaling cascades have been reported to be modulated by ROS [28]. The present study was designed to test the hypothesis that 915 MHz EMFs affect myocardial antioxidant capacity and apoptotic cell death in vitro and in vivo.

Potential harmful effects of EMF on biological systems have been considered controversial. Currently, there are debates about the influence of EMFs on DNA damage or oxidative stress. Indeed, some data have described damaging activities, conversely others have described no evident influence [29]. The observed bio-effects are dependent on various conditions, such as cells oxidative status, level of anti-oxidative enzymes, cell type, and parameters of the applied EMF [29]. Studies in vitro and in vivo revealed that EMFs at very low frequencies induced an increased production of free radicals, which can determine DNA double-strand breaks [29]. Interestingly, in vitro, in primary rat neocortical astroglial cell culture, 900 MHz induces ROS production and DNA damage [30]. Moreover, a range of studies have demonstrated that various cell lines do respond differently to EMF exposure, as, in vitro, six types of different cell lines exposed to EMF at 1800 MHz for 1 h and 24 h showed DNA damage in a cell type-dependent manner [31], while human glioblastoma cell lines exposed to 1950 MHz of EMF did not cause chromosomal damage after 50, 100, 150, and 200 h [32]. In addition, no significant ROS generation was measured in human cell lines exposed to 1800 MHz [33]. In accordance, our study showed no remarkably differences in oxidative status. Indeed, the short-term EMF-exposure of myocytes to 915 MHz did not significantly change the gene expression of antioxidant enzymes as compared to control conditions. Moreover, in vivo, the 1950 MHz EMF exposure did not affect oxidative stress in the aging brain [34]. On the contrary, a previous study showed that EMF induced oxidative damage and it was associated with neurodegenerative diseases with deleterious effects on brain tissue [35]. Another study reported that RF exposure at 1800 MHz slightly elevated the concentration of lipid peroxidation markers in the in brain, blood, liver, and kidney [36].

The activation of apoptosis is also considered to be involved in possible damage induced by EMF. An in vitro study reported that 1950 MHz signals induced apoptosis in astrocytes with the involvement of *Bax* and *Bcl2* [37]. In the present study, to elucidate whether EMF affected cell death in cardiac cells, we evaluated mRNA expression of *Bax* (pro-apoptotic factor) and *Bcl2* (anti-apoptotic factor) in H9C2 cells subjected to EMF 915 MHz for 24 h, 48 h, and 72 h. We did not observe significant differences in *Bax*, *Bcl2*, and *catalase* levels after short-term EMF exposure.

Exposure to EMFs can damage biological tissues by inducing changes in structure and function [1,4]. We provide in vivo evidence that 915 MHz of EMF exposure did not induce alterations in myocardial structure and apoptosis in mice at 48 h and 72 h. Furthermore, an analysis of antioxidant system capacity EMF-exposed mice demonstrated no significant alterations in the expression of antioxidant enzymes as compared to control mice. Thus, an in vivo study does not support the hypothesis that short-term exposure to 915 MHz promotes changes in the heart. However, we cannot rule out that long-term exposure to electromagnetic stress may be risk factors for myocardial damage.

## 5. Conclusions

Both in vitro and in vivo short-term studies found no evidence that EMFs affect antioxidant and apoptosis status in cardiac cells and tissues. Further studies examining dynamic changes in oxidative stress and apoptosis after long-term cardiac cell exposure to EMFs are warranted. These data provide an important reference in relation to the cellular antioxidant defense system and programmed cell death in response to electromagnetic stress.

## Data Availability

The data presented in this study are available on request from the corresponding author.

## Supplementary Materials

The following supporting information can be downloaded at: https://www.mdpi.com/article/10.3390/biomedicines10050929/s1, Table S1: List of primer sequences.
