# Supplementary material for: Cardiac Cell Exposure to Electromagnetic Fields: Focus on Oxdative Stress and Apoptosis
Source: Biomedicines. 2022 Apr 19;10(5):929. doi: 10.3390/biomedicines10050929 (PMC9138495; doi:10.3390/biomedicines10050929)
Supplement: Supplementary file 1 [file biomedicines-10-00929-s001.zip › biomedicines-1645225-supplementary.pdf]

Supplementary Table S1. Real-time PCR primer sequences.

Genes	Forward Sequence (5'-3')	Reverse Sequence (5'-3')
GAPDH	CTTTGTCAAGCTCATTTCCTGG	TCTTGCTCAGTGTCCTTGC
RPLP0	TGACATCGTCTTTAAACCCCG	TGTCTGCTCCCACAATGAAG
BAX	GGCGAATTGGAGATGAACTG	CCCCAGTTGAAGTTGCCAT
BCL2	GATGACTGAGTACCTGAACCG	CAGAGACAGCCAGGAGAAATC
CASPASE 3	GACTGATGAGGAGATGGCTTG	TGCAAAGGGACTGGATGAAC
CASPASE 8	AACTTCCTAGACTGCAACCG	TCTCAATTCCAACTCGCTCAC
CATALASE	GAACAGATGGCTTTTGACCC	GTAGGGACAGTTCACAGGTATC
SOD2	GGACAAACCTGAGCCCTAAG	CAAAAGACCCAAAGTCACGC
